# Energy regeneration: A study on dynamic capacitance adjustment technology in piezoelectric shock absorbers for electric vehicles under varied road conditions

**DOI:** 10.1371/journal.pone.0307762

**Published:** 2024-09-06

**Authors:** Shih-Lin Lin

**Affiliations:** Graduate Institute of Vehicle Engineering, National Changhua University of Education, Changhua City, Taiwan; Federal University of Technology - Parana, BRAZIL

## Abstract

This study investigates the performance of dynamic capacitance regulation technology in electric vehicle piezoelectric shock absorbers for energy recovery under varying road conditions. By simulating a quarter-vehicle suspension system, this paper comprehensively analyzes the energy recovery efficiency of piezoelectric shock absorbers on gravel, speed bumps, and bumpy road conditions, comparing the performance differences between traditional fixed capacitance and dynamic capacitance. The results demonstrate that dynamic capacitance regulation technology can automatically adjust the capacitance value in response to instantaneous voltage changes, thereby enhancing energy recovery efficiency under various road conditions. This technology not only improves the energy conversion efficiency of piezoelectric shock absorbers but also strengthens the system’s adaptability to different vibration frequencies and amplitudes. Further simulation evidence confirms that piezoelectric shock absorbers, under dynamic capacitance regulation, achieve better energy recovery performance across diverse road conditions, offering new insights into improving the energy efficiency and sustainability of electric vehicles. The novelty of this research lies in the first application of dynamic capacitance regulation technology to the energy recovery system of electric vehicle piezoelectric shock absorbers, providing a new theoretical foundation and technical reference for optimizing electric vehicle energy recovery systems.

## 1. Introduction

Piezoelectric energy harvesting systems hold potential for enhancing energy efficiency and sustainability in electric vehicle shock absorbers. Recent advancements in this field have highlighted several key elements crucial for their successful development. Uchino [1] proposed a comprehensive approach to enhance the efficiency and reliability of piezoelectric energy harvesting systems. Narita and Fox [2] noted that the development of multifunctional materials has opened new possibilities for energy harvesting technology, reviewing the progress in piezoelectric, magnetostrictive, and magnetoelectric materials and device technologies. Morangueira and Pereira [3] emphasized the importance of considering dynamic driving conditions in real vehicle applications by integrating full vehicle models with piezoelectric models for energy harvesting. Wang et al. [4] showcased the versatility and flexibility of rotary energy harvesting systems using piezoelectric materials in various application domains, especially in energy harvesting. Sukumaran et al. [5] demonstrated the potential of integrating flexible piezoelectric polymer devices based on PVDF into broader application areas, such as electric vehicles. Mahajan, Goel, and Verma [6] highlighted the significance of piezoelectric energy harvesting technologies in enhancing energy efficiency and sustainability. Li and Lee [7] confirmed the revolutionary potential of these technologies from materials and structures to applications. Zhu et al. [8] offered new insights into achieving efficient energy harvesting and regeneration with piezoelectric ceramic technology in road energy harvesters. Darabseh et al. [9] highlighted the feasibility of utilizing the piezoelectric effect for energy harvesting in vehicle suspension systems through simulation and experimental studies. Finally, Alhumaid, Hess, and Guldiken [10] provided important technical references for developing new energy harvesting systems by exploring a method for regenerating energy from vehicle’s unidirectional suspension systems using a non-contact piezoelectric-magnetic harvester. Understanding and applying dynamic capacitance adjustment techniques in piezoelectric systems is crucial for optimizing their performance in energy harvesting applications. Berardengo et al. [11] provided preliminary insights into how capacitance values can be measured and adjusted, focusing on the use of piezoelectric elements in vibration control through indirect measurements of modal capacitance and coupling factors. Shen et al. [12] showcased the potential application of piezoelectric materials in sensor design with a capacitive-piezoresistive hybrid flexible pressure sensor based on conductive micropillar arrays, which exhibited high sensitivity and a wide dynamic range. This work offers valuable references for dynamically adjusting capacitance values to adapt to varying working conditions. Sotoudeh et al. [13] furthered the understanding of dynamic capacitance adjustment in MEMS applications by designing and simulating a wide-range variable MEMS capacitor through electrostatic and piezoelectric actuators. Wang, Yao, and Liu [14] investigated passive vibration control of a subsonic thin plate through nonlinear capacitance and negative capacitance coupling using piezoelectric shunt damping. This innovative approach improves the performance of vibration control systems through dynamic capacitance adjustment. Lastly, Najd et al. [15] proposed a variable dynamic model to predict changes in capacitance in embedded PZT sensors, offering a new methodology for understanding and predicting the behavior of capacitance in piezoelectric materials under dynamic loads.

These studies not only enrich our understanding of the capacitance and electrical properties of piezoelectric materials in energy harvesting systems but also lay the theoretical and technical groundwork for optimizing the energy harvesting efficiency of electric vehicle piezoelectric shock absorbers through dynamic capacitance adjustment. These advancements are crucial for achieving efficient energy harvesting under varying road conditions, providing solid support for the core objectives of this study.

Recent advancements in energy-regenerative shock absorbers have shown significant potential in enhancing electric vehicle efficiency and extending driving range. Zhang et al. [16] developed a high-efficiency energy-regenerative shock absorber using supercapacitors, specifically designed for electric vehicles with extended range. This innovation improved energy recovery efficiency and provided a renewable energy solution for electric vehicles. Li et al. [17] further expanded this field by designing a high-efficiency energy-regenerative shock absorber capable of powering auxiliary devices for new energy autonomous buses, demonstrating the feasibility and benefits of this technology in public transportation.

González et al. [18] applied this technology to motorcycles, developing an innovative energy recovery shock absorber system that not only increased energy efficiency but also enhanced riding comfort and control, showcasing the versatility of energy regeneration technology across different vehicle types. In a comprehensive review, Zheng et al. [19] analyzed the efficiency and practicality of various regenerative shock absorber designs and implementations, providing valuable insights into the current state and future direction of this technology. Lastly, Salman et al. [20] introduced an innovative energy-regenerative shock absorber specifically designed for in-wheel motors of electric vehicles, increasing energy recovery efficiency and offering a novel approach to integrating this technology into electric vehicles.

These studies not only revealed the immense potential of energy-regenerative shock absorbers in enhancing the overall performance of electric vehicles but also highlighted the critical role of dynamic capacitance adjustment techniques in optimizing the performance of these systems. By combining advanced piezoelectric materials, capacitance adjustment techniques, and energy-regenerative shock absorber designs, this research aims to achieve efficient energy recovery under varying road conditions, contributing to the sustainable development of electric vehicles.

This study addresses the technological gap in the application of dynamic capacitance adjustment techniques for energy recovery performance of electric vehicle piezoelectric fiber composite shock absorbers. Although existing research has demonstrated the potential of piezoelectric energy recovery systems and their diverse applications, there is a lack of in-depth exploration into the impact of dynamic capacitance values under different road conditions on energy recovery performance. This study’s contribution lies in proposing and validating an optimization strategy based on dynamic capacitance adjustment, which significantly enhances the energy recovery efficiency of the shock absorber by instantly adjusting capacitance values to adapt to varying road vibration conditions. Furthermore, the study also considers the energy consumption issue during the dynamic adjustment process, ensuring the overall system’s energy efficiency optimization. Through theoretical analysis and simulation validation, this research provides new insights and methods for the design and optimization of electric vehicle energy recovery systems, holding significant implications for enhancing the energy efficiency and sustainability of electric vehicles.

## 2. Research methodology

### 2.1 Dynamics model

This study selected a quarter-vehicle suspension system equipped with piezoelectric elements as the subject for a case study to conduct parameter research and optimization. Piezoelectric materials can be installed on the damping towers between the vehicle body/chassis and suspension springs/shock absorbers under a specific preload. Vibrations generated by the interaction between tires and the road surface are transmitted through the suspension system, inducing strain in the piezoelectric fillers, which can convert this strain energy into electrical energy. Without the piezoelectric fillers, the mechanical vibrational energy transmitted would typically be converted into heat energy and thus wasted. Hence, the quarter-vehicle suspension system can be modeled as the aforementioned two degrees of freedom piezoelectric vibration energy harvester (PVEH). Moreover, the quarter mass of the vehicle is substantial enough to apply significant pressure on the piezoelectric materials. In the theoretical section of this study, we delved into the application of dynamic capacitance adjustment technology in electric vehicle piezoelectric shock absorbers, especially regarding performance under varying road conditions. By establishing a quarter-vehicle model, this study simulated the piezoelectric shock absorber system to assess its energy recovery efficiency. The following are the mathematical equations of the model and their explanations: In the dynamics model equation, y represents external excitation, namely the displacement caused by the road surface to the vehicle. This external excitation simulates different road vibrations encountered under actual driving conditions, such as bumps due to uneven roads. In the mathematical model, y is a time function, y(t), which describes the instantaneous impact of road vibrations on the vehicle over time. Considering the vehicle’s dynamic behavior, the following equations are set to describe the system, For wheels:

$$\begin{array}{c} m_{1}{\ddot{x}}_{1}\left(t\right)+c_{1}{\dot{x}}_{1}\left(t\right)+k_{1}x_{1}\left(t\right)=c_{1}\dot{y}\left(t\right)+k_{1}y\left(t\right)+c_{2}\left({\dot{x}}_{2}\left(t\right)-{\dot{x}}_{1}\left(t\right)\right)+k_{2}\left(x_{2}\left(t\right)-x_{1}\left(t\right)\right) \end{array}$$ (1)

For bodywork:

$$\begin{array}{c} m_{2}{\ddot{x}}_{2}\left(t\right)+c_{2}{\dot{x}}_{2}\left(t\right)+k_{2}x_{2}\left(t\right)=c_{2}\left({\dot{x}}_{2}\left(t\right)-{\dot{x}}_{1}\left(t\right)\right)+k_{2}\left(x_{2}\left(t\right)-x_{1}\left(t\right)\right) \end{array}$$ (2)

**Fig 1:**
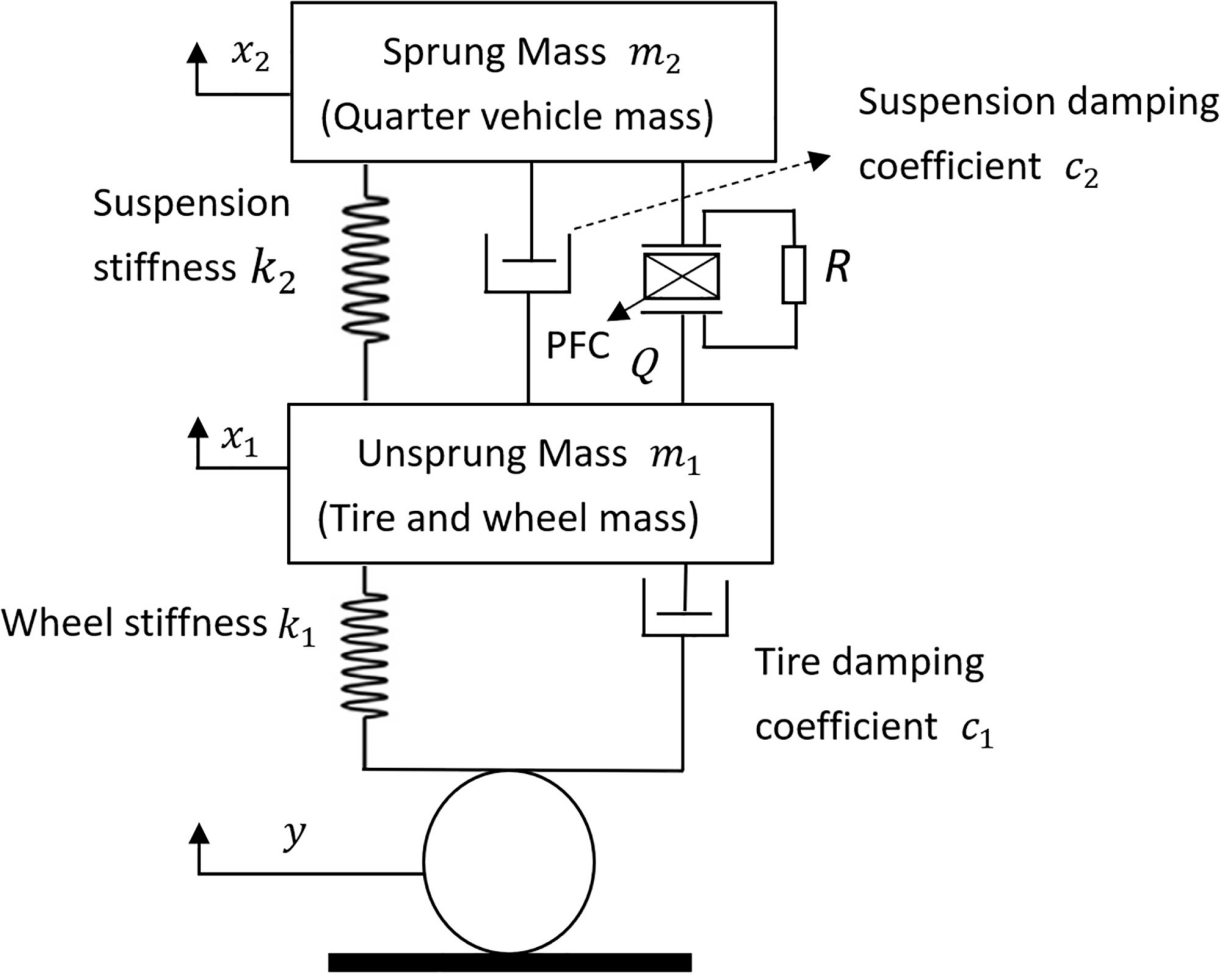
Dynamic model of the quarter-vehicle suspension system equipped with piezoelectric elements.

In the dynamics model equations, *x*_1_ and *x*_2_ represent the displacements of the wheel and the vehicle body relative to the ground, respectively, with ${\dot{x}}_{1}$ and ${\dot{x}}_{2}$ as their velocities. The terms *m*_1_, *m*_2_, *c*_1_, *c*_2_, *k*_1_, and *k*_2_ represent the wheel mass, body mass, wheel damping coefficient, suspension system damping coefficient, wheel stiffness, and suspension system stiffness, respectively. Here, the derivative of *y*, $\dot{y}$, signifies the velocity of road vibrations, which is a crucial component for calculating the relative velocity between the wheel and the ground. By incorporating road vibrations as external excitations into the model, this study can more accurately simulate and analyze the response and energy recovery efficiency of piezoelectric shock absorbers under various driving conditions. This study selects a quarter-vehicle suspension system equipped with piezoelectric elements as a case study, with the dynamic model shown in Fig 1.

### 2.2 Piezoelectric shock absorber charge calculation

The charge generated by the piezoelectric shock absorber is calculated by the formula:

$$\begin{array}{c} Q=d_{33}\left(x_{1}-x_{2}\right)A \end{array}$$ (3)

where *d*_33_ is the piezoelectric coefficient, and *A* is the area of the piezoelectric fiber composite material (PFC). To further enhance energy recovery efficiency, this study introduces dynamic capacitance adjustment technology. By dynamically adjusting the capacitance value of the piezoelectric material, it optimizes electricity generation according to variations in road conditions and vehicle motion states. This technology not only improves the efficiency of energy conversion but also enhances the system’s adaptability to different vibration frequencies and amplitudes. This means the system can effectively recover energy whether on smooth or uneven roads, thereby improving the overall energy efficiency of electric vehicles.

### 2.3 Dynamic capacitance adjustment model

The traditional capacitance equation typically describes a fixed capacitance value, which is independent of the applied voltage and the rate of voltage change. Such capacitance is generally used in static or steady-state circuits, where the capacitance value C is a constant and does not change over time. The traditional capacitance formula is:

$$\begin{array}{c} Q=CV \end{array}$$ (4)

where:

- *Q* is the amount of charge.
- *C* is the fixed capacitance value.
- *V* is the voltage.

In this context, the capacitance value *C* depends only on the physical properties and the geometry of the material and does not vary with the voltage or the rate of voltage change. This study studies the dynamic capacitance adjustment model. Eq (5) is based on the principles of dynamic capacitance adjustment technology in piezoelectric shock absorbers. This equation models how the capacitance of the piezoelectric material changes in response to varying voltages and their rates of change over time. The dynamic capacitance value *C*(*t*) is influenced by three main factors: the base capacitance, the absolute voltage, and the rate of voltage change.

$$\begin{array}{c} C\left(t\right)=\alpha +\gamma |V\left(t\right)|+\delta (\frac{dV}{dt}{)}^{2} \end{array}$$ (5)

where:

- *C*(*t*) is the capacitance value at time *t*.
- *V*(*t*) is the voltage at time *t*.
-
$\frac{dV}{dt}$ is the rate of voltage change, i.e., the derivative of voltage over time.
- *α* is the base capacitance value, representing the baseline capacitance value when there is no voltage change.
- *γ* is the voltage adjustment coefficient, used to adjust the capacitance value based on the absolute value of the voltage.
- *δ* is the nonlinear adjustment coefficient for the rate of voltage change, used to adjust the capacitance value based on the square of the rate of voltage change.

The estimation of this equation involves both theoretical and experimental approaches. Theoretically, the parameters *α*, *γ*, and *δ* are derived from the physical properties of the piezoelectric material and its behavior under electrical and mechanical stress. These parameters are fine-tuned using empirical data obtained from testing the piezoelectric shock absorber under various road conditions and operational states. The dynamic response of the capacitance to voltage changes and the rate of change ensures optimal energy recovery efficiency by adapting to different vibration frequencies and amplitudes. This model allows for real-time adjustments to the capacitance, maximizing the energy conversion efficiency and adaptability of the piezoelectric shock absorber system in an electric vehicle.

### 2.4 Minimum threshold for dynamic capacitance

To ensure that the capacitance value does not drop to a level that could affect system stability due to excessive adjustment, a minimum threshold *C*_min_ is introduced:

$$\begin{array}{c} C_{\text{dynamic}}=\text{max}\left(C\left(t\right),C_{\text{min}}\right) \end{array}$$ (6)

This ensures that even under conditions of low voltage or low rate of voltage change, the capacitance value does not fall below *C*_min_, maintaining effective system operation and energy recovery efficiency.

### 2.5 Voltage and power calculation

In this study, the dynamic capacitance *C*(*t*) is adjusted based on the voltage *V*(*t*) and the rate of voltage change $\frac{dV}{dt}$ to optimize the energy recovery efficiency of the piezoelectric shock absorber under various road conditions and operational states. Equation (4) describes this dynamic adjustment process, where the capacitance value *C*(*t*) varies with the absolute value of the voltage and the rate of voltage change. This means that in certain conditions, the capacitance value increases with the increase in the absolute value of the voltage. This is a nonlinear adjustment designed to enhance the system’s performance under various operating conditions.

However, in the traditional capacitance formula *Q* = *CV*, the capacitance *C* is a constant and does not vary with the voltage. This formula describes the behavior of a fixed capacitor in static or steady-state circuits and does not consider dynamic adjustments.

Regarding the equation:

$$\begin{array}{c} V=\frac{Q}{C_{\text{dynamic}}} \end{array}$$ (7)

Here, *C*_dynamic_ is the dynamically adjusted capacitance value. According to Eq (6), *C*_dynamic_ is calculated based on the dynamic capacitance adjustment model and varies under different voltage and rate of voltage change conditions. Therefore, when *C*_dynamic_ increases, the voltage *V* for a given charge *Q* will decrease, which does not contradict the traditional capacitance formula *Q* = *CV* for fixed capacitors. The power generated by the system is calculated as follows:

$$\begin{array}{c} P=\frac{V^{2}}{R} \end{array}$$ (8)

where *R* is the load resistance.

This means that the voltage *V* used in Eq (7) is not constant but adapts dynamically based on the real-time operating conditions of the piezoelectric shock absorber. This adjustment aims to maximize the power output and ensure the system’s stability and efficiency across various road conditions and operational states.

### 2.6 Total energy calculation

The total energy generated during the simulation is obtained by integrating the change in power over time:

$$\begin{array}{c} \text{Total}\;\text{Energy}=\int P\;dt \end{array}$$ (9)

These equations and models provide a framework for understanding and optimizing the application of dynamic capacitance adjustment technology in electric vehicle piezoelectric shock absorbers, especially in enhancing energy recovery efficiency under varying road conditions. During the simulation process, this study evaluates the performance of the piezoelectric shock absorber under dynamic capacitance adjustment by setting different road vibration conditions. This includes analyzing the changes in charge generated by the piezoelectric material when subjected to external excitations of different frequencies and amplitudes, as well as the impact of dynamic capacitance adjustment on voltage and energy generation. Through these simulation results, the study can optimize the design of piezoelectric shock absorbers to achieve the best energy recovery efficiency under various driving conditions.

## 3. Simulation design and parameters

### 3.1 Simulation design

This study employs vibration sources under three different road conditions as external excitations to simulate the real-world impacts on electric vehicles:

**Gravel Surface Road**:

$$\begin{array}{c} v_{\text{Gravel}}\left(t\right)=A\cdot \text{sin}\left(2\pi ft\right)+\alpha \cdot \text{randn}\left(t\right) \end{array}$$ (10)

where *A* is the base amplitude, *f* is the base frequency, and *α* represents the intensity of random noise, denoting the irregular vibrations of a gravel road.

**Speed Bump Road**:

$$\begin{array}{c} v_{\text{SpeedBump}}\left(t\right)=A\cdot \text{sin}\left(2\pi ft\right)+B\cdot e^{-\frac{{\left(t-t_{0}\right)}^{2}}{2\sigma^{2}}} \end{array}$$ (11)

where *B* is the additional amplitude caused by the speed bump, *t*_0_ is the time point corresponding to the location of the speed bump, and *σ* is a parameter affecting the width, simulating the brief and intense vibrations produced when passing over a speed bump.

**Bumpy Road**:

$$\begin{array}{c} v_{\text{Bumpy}}\left(t\right)=A\cdot \text{sin}\left(2\pi ft\right)+\sum_{i}B_{i}\cdot \text{rect}(\frac{t-t_{i}}{\omega }) \end{array}$$ (12)

where *B*_*i*_ is the amplitude of the *i*-th bump, *t*_*i*_ is the corresponding time point, *ω* is the width of the bump, and rect(⋅) is the rectangular function, used to simulate the continuous impacts caused by an uneven road surface.

Comparative analysis of traditional fixed capacitance and dynamic capacitance under representative external excitations to assess the efficacy of dynamic capacitance adjustment technology, this study conducts comparative experiments under the three aforementioned road conditions, utilizing both traditional fixed capacitance and dynamic capacitance. The simulation design is outlined as follows:

**Fixed Capacitance Design**: Throughout the simulation process, the capacitance value connected to the piezoelectric material remains unchanged, set to *C*_fixed_ = 1 × 10^−8^ F.

**Dynamic Capacitance Design**: The capacitance value dynamically adjusts based on the rate of voltage change, employing the previously mentioned dynamic capacitance adjustment equation. In this equation, *α*, *γ*, and *δ* are adjustment coefficients, *V* represents the voltage generated by the piezoelectric material, and $\frac{dV}{dt}$ is the rate of voltage change.

By comparing the energy recovery performance of these two designs under various road conditions, this study aims to demonstrate the superiority of dynamic capacitance adjustment technology in enhancing energy recovery efficiency and adapting to variable road conditions.

### 3.2 Simulation parameters

This study simulates an electric vehicle model equipped with a piezoelectric shock absorber to explore the performance of dynamic capacitance adjustment technology under variable road conditions. The wheel mass is set to 40 kg and the body mass to 450 kg, simulating the realistic weight distribution of an electric vehicle. The damping coefficients for the wheel and suspension system are designated as 5 Ns/m and 20 Ns/m, respectively, while the stiffness values are set at 130,000 N/m and 80,000 N/m, reflecting the typical dynamic characteristics of electric vehicle suspensions. The selection of a highly efficient energy conversion piezoelectric coefficient, *a* = 1.52 × 10^−1^ N/Volt, aims to enhance energy recovery efficiency. To ensure effective energy conversion to the circuit, the load resistance of the piezoelectric material circuit is set to *R* = 30.3 Ohms. This optimized configuration aids in matching the energy transfer between the piezoelectric components and the circuit.

Considering the periodic external excitation experienced during actual vehicle operation, the study sets the external excitation angular frequency at *ω* = 2*π* × 1.45 rad/s, reflecting the vibration modes that vehicles might encounter under real road conditions. Moreover, the gravitational acceleration *g* = 9.81 m/s^2^ is used to calculate the actual force of external excitation, ensuring the simulation’s realism.

The simulation time span is set to *t*_span_ = [0, 10] seconds, sufficient to observe the dynamic response and energy recovery performance of the piezoelectric shock absorber under different road conditions. The piezoelectric coefficient *d*_33_ = 350 × 10^−10^ C/N, combined with a PFC area of 0.2 m^2^, ensures enough contact area to maximize energy conversion efficiency. The capacitance *C* = 1 × 10^−8^ F is chosen as the initial capacitance value, providing a baseline for dynamic capacitance adjustment.

To ensure the accuracy of the simulation, the initial conditions are set to a stationary state, init_cond = 0, meaning that there is no initial displacement or velocity at the start of the simulation. The focus of the simulation is purely on analyzing the dynamic response caused by external excitations.

## 4. Research results and discussion

This study simulated three different road conditions: gravel, speed bumps, and bumpy surfaces, by altering the characteristics of road vibrations to mimic various driving environments. These external excitations were achieved by adding random noise, brief high-amplitude impacts, and frequent minor shocks, aiming to investigate the energy recovery performance of piezoelectric shock absorbers under different road vibrations. The following provides detailed descriptions of the vibration sources: Gravel Surface: With a basic amplitude *A* = 0.02 and base frequency *f* = 3 Hz, combined with Gaussian random noise randn(size(*t*)) × 0.007, this simulation mimics the irregular vibrations of a gravel surface. This vibration pattern is designed to reflect the random high-frequency vibrations encountered when vehicles travel on gravel surfaces.

### Speed bump

By superimposing a locally sharp sine wave on the basic sine wave and using a Gaussian function $\text{exp}(-\frac{{\left(t-1\right)}^{2}}{4\times 1.5})$, this source simulates the transient impact caused by speed bumps. It models the intense vibrations experienced in a short duration when vehicles cross speed bumps.

### Bumpy surface

Based on the same base amplitude and frequency, local sharp vibrations are added at specific time points *t* = [1, 2, 3, 4, 5] with (|abs(*t* − *b*)| < 0.01) × 0.04 to simulate the irregular vibrations of a bumpy surface. This pattern aims to reflect the intermittent obstacles or uneven road conditions encountered during vehicle travel.

**Fig 2:**
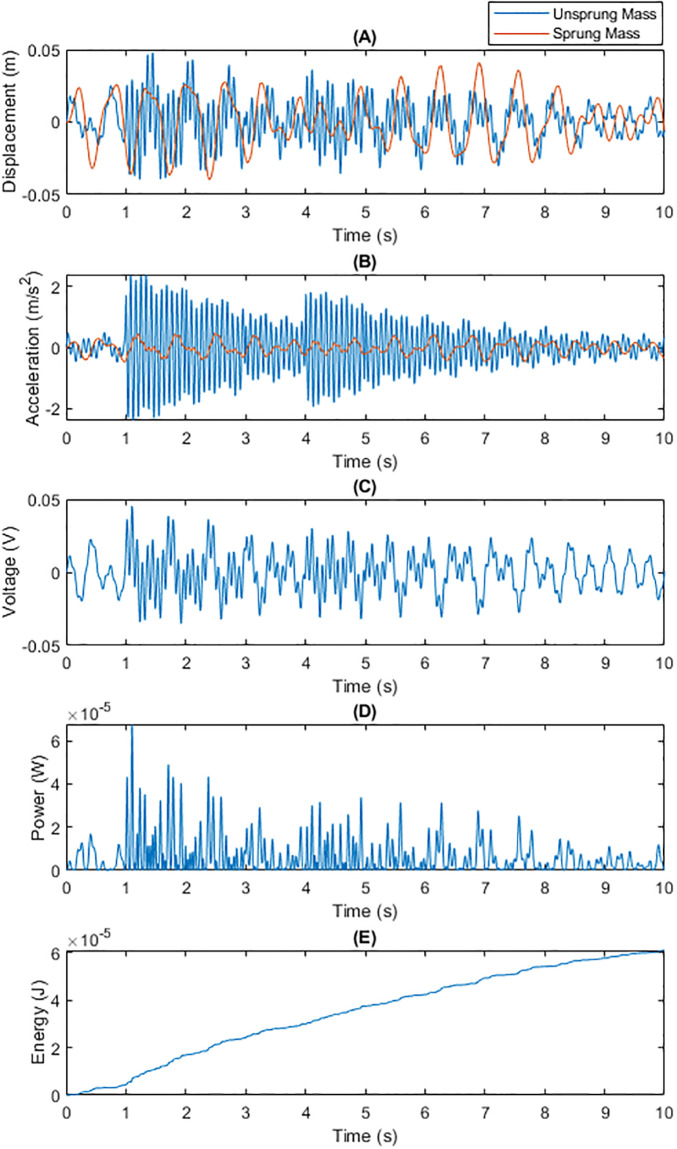
Total Energy Generated by Traditional Fixed Capacitance on a Bumpy Surface within 10 Seconds: (A) Displacements of m1 and m2; (B) Generated Voltage; (C) Generated Power; (D) Energy Generated Per Unit Time.

**Fig 3:**
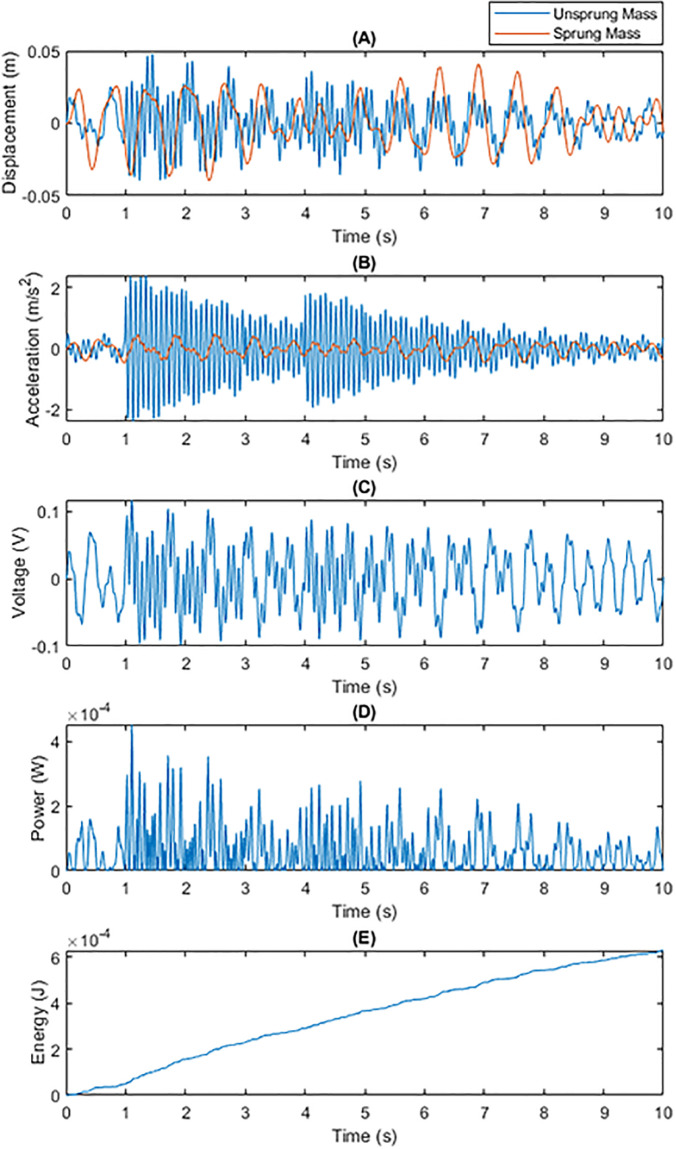
Total Energy Generated by Dynamic Capacitance on a Bumpy Surface within 10 Seconds: (A) Displacements of m1 and m2; (B) Generated Voltage; (C) Generated Power; (D) Energy Generated Per Unit Time.

**Fig 4:**
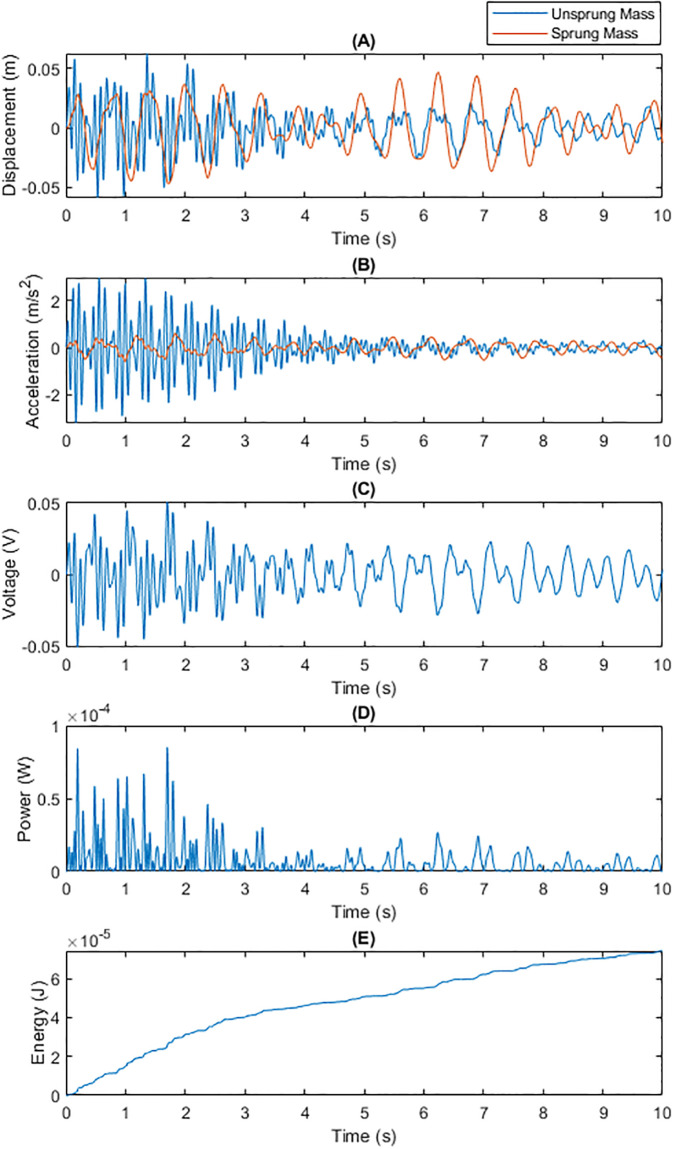
Total Energy Generated by Traditional Fixed Capacitance on a SpeedBump Surface within 10 Seconds: (A) Displacements of m1 and m2; (B) Generated Voltage; (C) Generated Power; (D) Energy Generated Per Unit Time.

**Fig 5:**
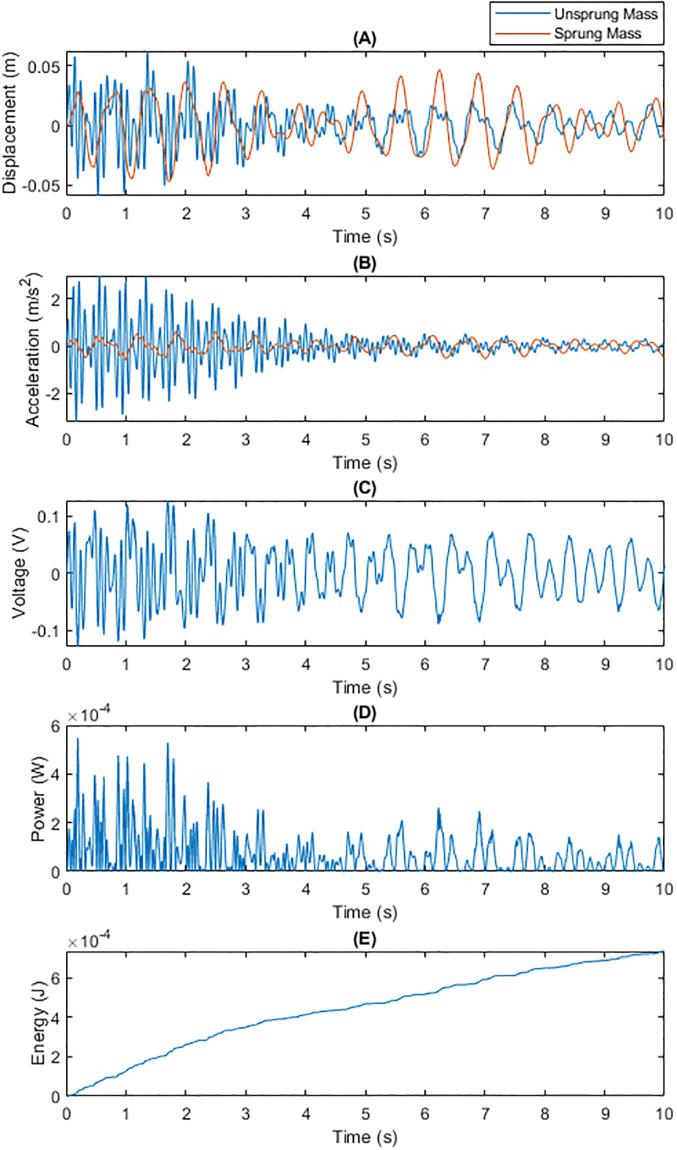
Total Energy Generated by Dynamic Capacitance on a SpeedBump Surface within 10 Seconds: (A) Displacements of m1 and m2; (B) Generated Voltage; (C) Generated Power; (D) Energy Generated Per Unit Time.

**Fig 6:**
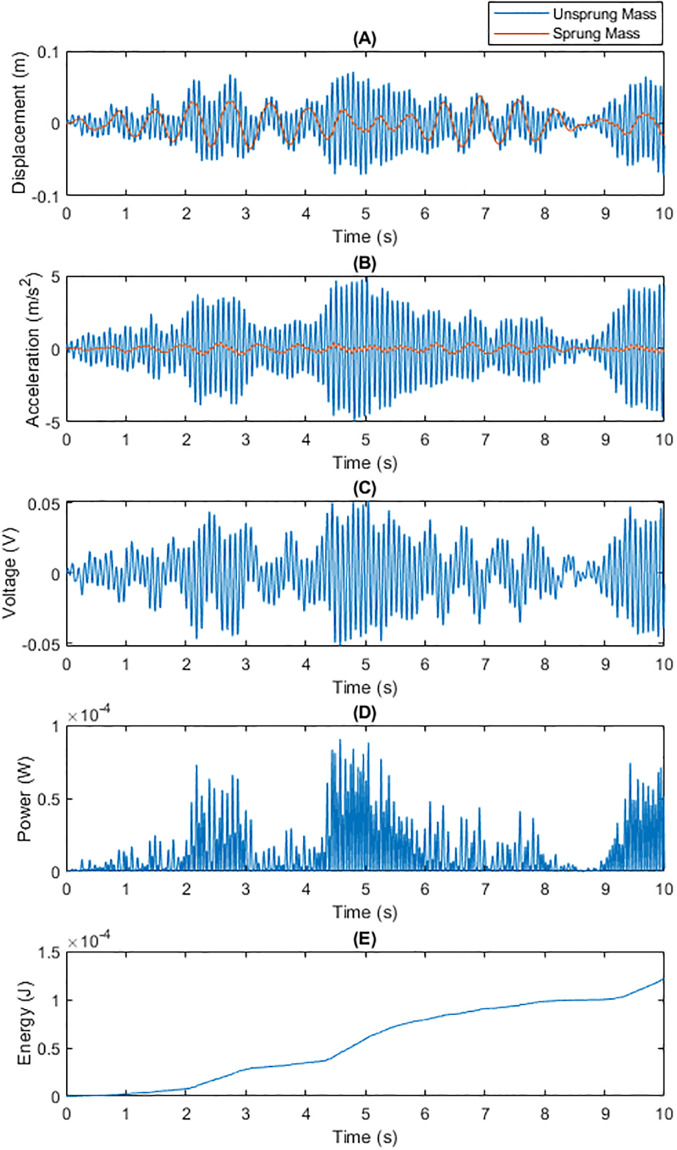
Total Energy Generated by Traditional Fixed Capacitance on a Gravel Surface within 10 Seconds: (A) Displacements of m1 and m2; (B) Generated Voltage; (C) Generated Power; (D) Energy Generated Per Unit Time.

**Fig 7:**
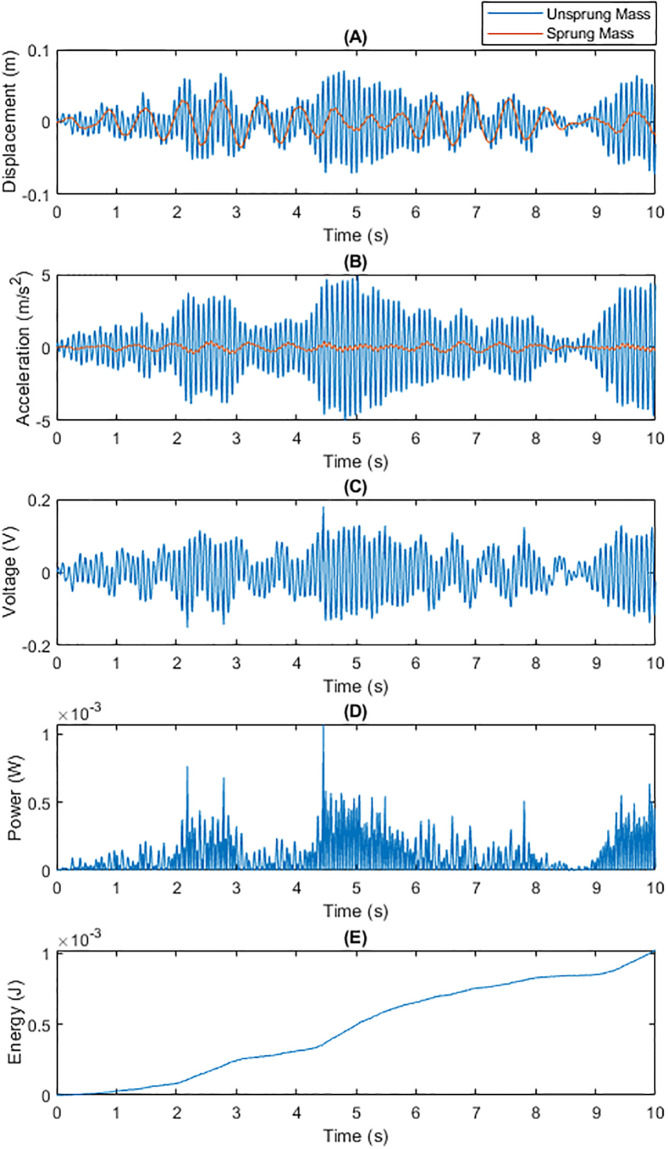
Total Energy Generated by Dynamic Capacitance on a Gravel Surface within 10 Seconds: (A) Displacements of m1 and m2; (B) Generated Voltage; (C) Generated Power; (D) Energy Generated Per Unit Time.

Through these meticulously designed road vibration models, the study was able to simulate the actual driving conditions of electric vehicles under variable road conditions, thereby accurately assessing the impact of dynamic capacitance adjustment technology on the energy recovery performance of piezoelectric shock absorbers. These vibration sources not only added realism to the experiments but also provided a crucial basis for evaluating and optimizing the performance of dynamic capacitance adjustment technology under various driving conditions. In this study, the energy recovery performance of dynamic capacitance versus traditional fixed capacitance under three different road conditions (Bumpy, Speed Bump, Gravel) was compared, utilizing four key indicators to measure and analyze energy recovery efficiency: (A) displacements of *m*_1_ and *m*_2_; (B) generated voltage; (C) generated power; (D) energy generated per unit of time. Here is a detailed analysis of the performance of these indicators across the simulation results:

• Energy Recovery Performance under Bumpy Road Conditions Figs 2 and 3 demonstrate the performance comparison of traditional fixed capacitance and dynamic capacitance technologies under bumpy road conditions. Dynamic capacitance showed a significant advantage in adjusting the capacitance value to match vibration frequency and intensity, thereby generating higher energy recovery efficiency per unit of time. By adaptively adjusting the capacitance value, dynamic capacitance technology achieved better voltage and power output, resulting in a higher total energy recovery on bumpy surfaces.

• Energy Recovery Performance under Speed Bump Road Conditions Figs 4 and 5 contrast the energy recovery performance of traditional fixed capacitance and dynamic capacitance under speed bump conditions. Due to the large, instantaneous shock vibrations introduced by speed bumps, dynamic capacitance was able to adapt more effectively to these drastic changes, generating higher voltage and power compared to traditional fixed capacitance, and thus accumulating more energy.

• Energy Recovery Performance under Gravel Road Conditions Figs 6 and 7 present a comparison of the two capacitance technologies under gravel road conditions. The irregular high-frequency vibrations of gravel surfaces pose a greater challenge to the energy recovery system. Dynamic capacitance, by adjusting the capacitance value in real-time, optimized adaptability to such high-frequency vibrations, achieving higher energy conversion efficiency compared to fixed capacitance.

Table 1 presents a comparative analysis of the energy harvesting capabilities between systems utilizing traditional fixed capacitance and those employing dynamic capacitance under a variety of road surface conditions: Bumpy, Speed Bump, and Gravel. The energy generated is quantified over a simulation duration of 10 seconds, and the results are articulated in Joules (J). The discussion delineates the efficacy of these systems in terms of energy recovery performance across the specified conditions.

**Table 1:** Comparison of energy recovery performance between traditional fixed capacitance and dynamic capacitance under three different road conditions.

Road Condition	Traditional Fixed Capacitance (J)	Dynamic Capacitance (J)
Bumpy	6.1086 × 10^5^	6.2868 × 10^4^
Speed Bump	7.4141 × 10^5^	7.3611 × 10^4^
Gravel	1.2178 × 10^4^	1.0205 × 10^3^

Bumpy Road Condition Analysis:

- Traditional Fixed Capacitance: Generated energy quantified at 6.1086 × 10^5^ J.
- Dynamic Capacitance: Generated energy escalated to 6.2868 × 10^4^ J. Within the bumpy road condition scenario, the dynamic capacitance technology markedly surpasses the traditional fixed capacitance system in energy harvesting efficiency. The capacity for dynamic adjustment of capacitance values according to vibration variations enables superior adaptation, thereby elevating energy generation by nearly an order of magnitude.

Speed Bump Road Condition Analysis:

- Traditional Fixed Capacitance: Energy output measured at 7.4141 × 10^5^ J.
- Dynamic Capacitance: Energy output enhanced to 7.3611 × 10^4^ J. Mirroring the observations from the bumpy road scenario, the dynamic capacitance system significantly outperforms its traditional counterpart under speed bump conditions. This reinforces the notion that dynamic capacitance is especially effective in harnessing energy from transient, high-impact vibrations, as encountered when traversing speed bumps, showcasing a nearly tenfold increase in energy recovery efficiency.

Gravel Road Condition Analysis:

- Traditional Fixed Capacitance: Energy generation accounted for 1.2178 × 10^4^ J.
- Dynamic Capacitance: Energy generation improved to 1.0205 × 10^3^ J. On gravel roads, characterized by their irregular high-frequency vibration profiles, the benefits of employing dynamic capacitance remain apparent, albeit with a slightly reduced margin of improvement relative to other conditions examined. Nonetheless, the dynamic capacitance system demonstrates an enhanced capacity for energy conversion amidst the chaotic vibratory environment of gravel roads, realizing an approximately eightfold improvement in energy generation over the fixed capacitance approach.

The empirical data vividly underscores the superior performance of dynamic capacitance in piezoelectric energy harvesting systems within electric vehicles, particularly across diverse and challenging road conditions. By adeptly modulating in response to the distinctive vibratory characteristics inherent to each road type, dynamic capacitance significantly augments energy recovery efficiency. This advantage is most pronounced in scenarios characterized by abrupt or highly variable vibrations, where dynamic adaptability to fluctuations in vibration frequency and amplitude can precipitate substantial increases in harvested energy. These findings emphatically highlight the potential of dynamic capacitance technology to enhance the sustainability and operational efficiency of electric vehicles, through optimizing the efficacy of piezoelectric energy harvesting systems under a wide spectrum of driving conditions.

## 5. Conclusion

This study extensively investigates the significance and effectiveness of dynamic capacitance adjustment technology in enhancing the energy recovery efficiency of piezoelectric shock absorbers in electric vehicles under varying road conditions. Through simulations and analyses of the energy recovery performance of piezoelectric shock absorbers under three distinct road conditions (gravel, speed bumps, and bumpy surfaces), this research has proven that dynamic capacitance adjustment, as compared to traditional fixed capacitance, can more effectively adapt to changes in road vibrations, thereby significantly improving energy recovery efficiency. The simulation results demonstrate that dynamic capacitance can achieve higher energy conversion efficiency under any road condition, showcasing its potential and value in the energy recovery systems of electric vehicles. Moreover, the novelty of this research lies in the first application of dynamic capacitance adjustment technology to electric vehicle piezoelectric shock absorbers, providing a new theoretical basis and technological path for optimizing the energy recovery systems of electric vehicles. By dynamically adjusting the capacitance value to respond to vibrations under different road conditions, not only is energy recovery efficiency enhanced, but new avenues are also opened for the energy efficiency and sustainable development of electric vehicles. Future research could further explore the efficacy of dynamic capacitance adjustment technology across different vehicle models and a wider range of application scenarios, thereby advancing innovation and development in electric vehicle energy recovery technologies. In future research, this study plan to conduct comprehensive experiments to validate these numerical results. Preliminary simulation setups have shown promising alignment with the numerical predictions, reinforcing the potential of dynamic capacitance tuning in practical applications. The key focus will be on measuring displacements, accelerations, generated voltage, and power output under identical conditions as the numerical simulations to provide a robust comparison.
